# Marginalisation and cardiovascular disease among rural Sami in Northern Norway: a population-based cross-sectional study

**DOI:** 10.1186/1471-2458-13-522

**Published:** 2013-05-29

**Authors:** Bent-Martin Eliassen, Marita Melhus, Ketil Lenert Hansen, Ann Ragnhild Broderstad

**Affiliations:** 1 Centre for Sami Health Research, Department of Community Medicine, Faculty of Health Sciences, University of Tromsø, N-9037, Tromsø, Norway; 2 Department of Medicine, University Hospital of Northern Norway, N-9480, Harstad, Norway

**Keywords:** Cardiovascular disease, Marginalisation, Stress, Indigenous, Sami, SAMINOR, Norway

## Abstract

**Background:**

Like other indigenous peoples, the Sami have been exposed to the huge pressures of colonisation, rapid modernisation and subsequent marginalisation. Previous studies among indigenous peoples show that colonialism, rapid modernisation and marginalisation is accompanied by increased stress, an unhealthy cardiovascular risk factor profile and disease burden. Updated data on the general burden of cardiovascular disease among the Sami is lacking. The primary objective of this study was to assess the relationship between marginalisation and self-reported lifetime cardiovascular disease (CVD) by minority/majority status in the rural Sami population of Norway.

**Methods:**

A cross-sectional population-based study (the SAMINOR study) was carried out in 2003-2004. The overall participation rate was 60.9% and a total of 4027 Sami individuals aged 36-79 years were included in the analyses. Data was collected by self-administrated questionnaires and a clinical examination.

**Results:**

The logistic regression showed that marginalised Sami living in Norwegian dominated areas were more than twice as likely to report CVD as non-marginalised Sami living in Sami majority areas (OR 2.10, 95% CI: 1.40-3.14). No sex difference was found in the effects of marginalisation on self-reported life-time cardiovascular disease. Moderate to no intermediate effects were seen after including established CVD risk factors.

**Conclusions:**

This study showed that marginalised Sami living in Norwegian dominated areas were more than twice as likely as non-marginalised Sami from Sami majority areas to report lifetime cardiovascular disease (CVD). Moderate to no intermediate effects were seen after including established CVD risk factors, which suggest little difference in lifestyle related factors. Chronic stress exposure following marginalisation may however be a plausible explanation for some of the observed excess of CVD.

## Background

The Sami are an indigenous people whose traditional settlement region (Sápmi) stretches across the northern parts of Norway, Sweden and Finland, and Russia’s Kola Peninsula. The Sami languages belong to the Finno-Ugric language group. The Sami settlement area of Norway is characterised by variety in the Sami population structure, language situation and traditions.

Like other indigenous peoples, the Sami have been exposed to the huge pressures of colonisation, ethnic discrimination, rapid modernisation and subsequent marginalisation. Thus, the Sami constitute a minority in most areas and a majority in a few. Marginalisation for many indigenous individuals involves amongst other things social exclusion, discrimination, and loss of indigenous language, traditions and subsistence rights [1-3]. Previous studies among indigenous peoples show that colonialism, rapid modernisation and subsequent marginalisation is accompanied by increased chronic stress, an unhealthy cardiovascular risk factor profile and disease burden [2]. No studies have been done on the effects of marginalisation on cardiovascular disease in the Sami population of Norway. High levels of diabetes and obesity have been documented [4,5] but updated data on the general burden of cardiovascular disease (CVD) is lacking.

The primary objective of this study was to assess the relationship between marginalisation and self-reported lifetime cardiovascular disease (CVD) by minority/majority status in the rural Sami population of Norway. If differences were found, we aimed at evaluating the role of established cardiovascular risk factors and educational attainment as mediating and confounding factors.

## Methods

As part of the Norwegian Institute of Public Health’s (NIPH) health survey in Finnmark, the Centre for Sami Health Research conducted in 2003-2004 a population-based survey in areas with Sami and non-Sami populations (SAMINOR) [6]. All eligible residents in the selected municipalities registered in the Central Population Register were invited regardless of ethnic background. In the age group 36-79 years 27,151 were invited; of these, 16,538 (60.9%) participated. The population was almost exclusively rural. The Regional Committee for Medical and Health Research Ethics of Northern Norway (REC North) [7], approved the study and the participants gave written informed consent.

Included in the analysis were respondents aged 36-79 years (born 1925-1968) who reported at least one Sami identity mark (n=5796), completed three questionnaires (initial, main and additional) (n=4380) and answered at least two of the three questions concerning marginalisation (n=4118). The questionnaires are attached as Additional files 1 and 2 at the end of this document and may also be accessed on the project website [8,9]. Furthermore, Sami participants living in Narvik and the south Sami area were not included due to small numbers (n=91). The total numbers for the subsequent analysis were 4027 Sami individuals (2013 women and 2014 men).

The self-administrated questionnaires were not validated in this population but most of the questions have been used in previous population studies in Norway. Ethnicity was ascertained by the following: *What language do/did you, your parents and your grandparents use at home?* Possible response options were “Norwegian”, “Sami”, “Kven”, “other”. Kvens are descendant of Finnish settlers immigrating to northern Norway in the 1700s and 1800s [10]. Providing the same answer categories we also asked: *What is your, your father’s and your mother’s ethnic background?* The respondents also reported whether they considered themselves to be Norwegian, Sami, Kven or other (self-perceived ethnicity). On all these questions multiple answers were allowed. Sami participants were identified if they reported at least one Sami identity mark (Sami language spoken by the respondent or at least one parent or grandparent, or Sami ethnic background or self-perceived Sami ethnicity). These variables are described in detail by Lund et al. [6].

Lifetime cardiovascular disease was measured by three questions: *Do you have, or have you had*: “Myocardial infarction (heart attack)? (Yes/No)”, “Angina pectoris (heart cramp)? (Yes/No)”, or “Cerebral stroke/brain haemorrhage (Yes/No)”? Missing values were considered negative responses.

Marginalisation was measured by asking the following three questions: *Do you feel you are being forced from your work/trade?*: “To a large extent”, “To some extent”, “To a small extent”, “Absolutely not”. Using the same response options, the second question was: *Do you feel that modern development displaces the Sami culture?* The third and final question was: *Have you experienced bullying/discrimination due to your ethnic (Sami, Kven, Russian, Tamil, Norwegian, etc.) background?*: “Many times”, “Sometimes”, “Rarely”, “Never”. The factor structure of these variables has in a prior study been assessed by using principal component analysis; Hansen [3] found a clustering of these items suggesting that the questions are measuring the same construct, i.e. *Feeling of marginalisation*. After recoding, the items were combined in a score ranging from 0–9 which based on its distribution was dichotomised into “unexposed to marginalisation” (scores 0–3) and “exposed to marginalisation” (scores 4–9). Missing values were given the value “0” (null), provided that information was given on at least two of the three variables. Where the information supplied was from fewer than two variables, the data were coded as missing.

Education level was based on the question: *How many years of schooling/education have you completed*? Years of education was dichotomised into 0-12 years and 13 years or more.

Data on smoking was collected by asking: *Are you currently, or were you previously a daily smoker*: “Yes, currently”, “Yes, previously,” “Never”? Those reporting never or current smoking were coded as past smokers given that they also reported number of years since they stopped smoking. Sensitivity analyses with pack-years in current smokers and time since smoking cessation in previous smokers were conducted.

The following two questions measured family history of CVD: *Tick off relatives who have, or have ever had, any of the following conditions, and report the age of when they got the illness*: “Myocardial infarction before the age of 60 years” or “Cerebral stroke” The possible answer categories were: “Mother”, “Father”, “Sister”, “Brother”, “Children”, “No one”. For myocardial infarction, observations providing positive answers but reporting the age ≥60 for when their relative(s) got the disease (n=78), were given a negative response. Those giving a negative response but reporting age <60 were given a positive response (n=2). For stroke observations providing a negative answer but reporting the age for when their relative(s) got the disease were given a positive response (n=2).

World Health Organization (WHO) define the metabolic syndrome as the presence of central obesity (waist circumference ≥102 cm in men and ≥88 cm in women) plus any two of four additional factors: Raised triglyceride level > 1.7 mmol/l, reduced HDL cholesterol < 1.03 mmol/l in males and < 1.29 mmol/l in females, raised blood pressure (systolic BP ≥ 130 mm Hg or diastolic BP ≥ 0.85 mmHg) and raised fasting plasma glucose ≥ 5.6 mmol/l (or previously diagnosed type 2 diabetes). All blood samples in the SAMINOR study were non-fasting. We used self-reported diabetes or information about anti-diabetic medication to define diabetes. Additionally, non-fasting blood glucose level > 11.1 was defined as having diabetes [11]. As the ability of MetS to predict cardiovascular disease is somewhat debated [12,13], we performed sensitivity analyses by including its components separately together with total cholesterol in the analysis.

Waist circumference was measured at the umbilicus to the nearest centimetre with the individual standing and breathing normally. The methods used and procedures followed for the measurement of blood pressure, total cholesterol, HDL cholesterol, triglycerides and glucose are described in detail elsewhere [6,14].

**Figure 1:**
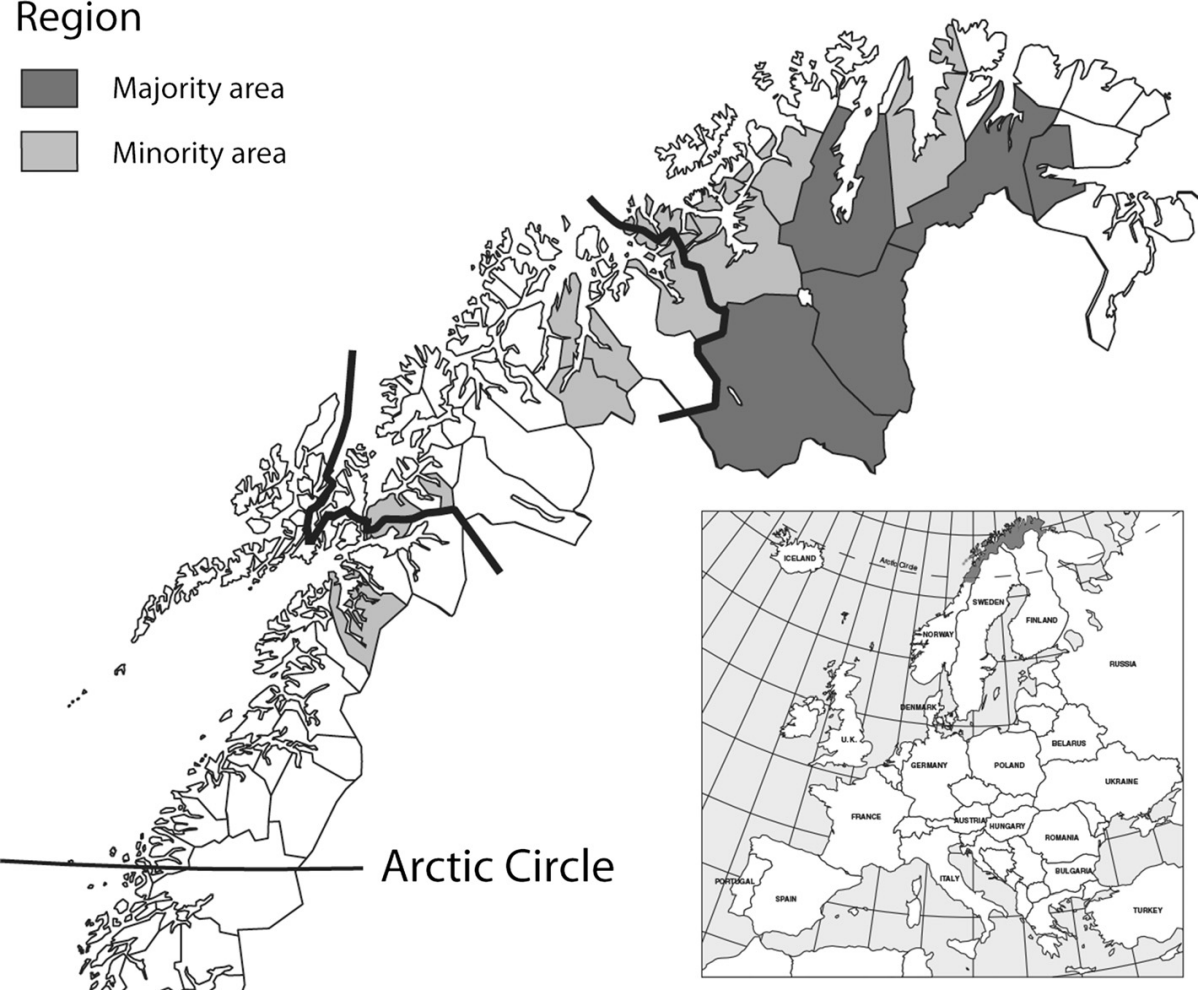
Municipalities included in the study.

We stratified our analysis geographically by Sami majority and minority areas. We defined the municipalities of Kautokeino, Karasjok, Nesseby, Tana and Porsanger as Sami majority areas, and the costal situated municipalities of Tysfjord, Evenes, Skånland, Lavangen, Lyngen, Storfjord, Kåfjord, Kvænangen, Alta, Loppa, Kvalsund, and Lebesby as Sami minority areas (Figure 1). Characteristic of the former are a majority Sami population in general (Kautokeino, Karasjok, Nesseby and Tana) or in certain areas of the municipality (Porsanger) and long-running schemes promoting Sami language, culture and primary industries. Furthermore, these areas are important reindeer husbandry districts. Tana, Nesseby and Porsanger also have a large coastal Sami population but these coastal areas are distinguishable from the other coastal Sami municipalities as they have a relatively large proportion of individuals reporting Sami ethnicity (data not shown).

We further stratified the analyses by marginalisation status (exposed/unexposed), thus ending up with four categories: “**unexposed** settled in **majority** area”, “**exposed** settled in **majority** area”, “**unexposed** settled in **minority** area” and “**exposed** settled in **minority** area”.

### Statistical analyses

The age-adjusted prevalence rates presented in Tables 1, 2 and 3 were based on logistic regression estimates. Differences between these rates were tested by performing likelihood ratio tests based on the same regression models. Age-specific and total crude rates in Table 3 were tested by Fisher’s exact chi-square tests. Sensitivity analyses on the cardiovascular disease rates in Tables 1, 2 and 3 were done by performing direct standardisation using the European population [15] as reference and comparing 95% CIs. Logistic regression (Table 4) was then used to evaluate age, education and established CVD risk factors as potential confounding or mediating factors in the association between marginalisation and Sami majority/minority areas, and CVD. The full model was assembled by including all covariates as specified in the footnote in Table 4. Backward regression was performed to assess possible intermediate effects by step-wise removal of the respective covariates and evaluating the changes in the effect of the main exposure on CVD. We did not perform sex-specific analysis in Table 4 as sensitivity analysis showed no sex effect (data not shown). We also did sensitivity analyses by including in turn the individual components of the MetS and total cholesterol, intensity and duration of smoking, and alcohol consumption in the full model and evaluating relevant point estimates, CIs and p-values.

**Table 1:** **Age-adjusted characteristics**^**a **^**of the female study group by majority/minority area and marginalisation status**

	**Unexposed (n=678)**			**Exposed (n=340)**			
**Majority**	**Tot**	**n**	**%**	**Tot**	**n**	**%**	**P**^**b**^
Myocardial infarction	678	9	1.3	340	1	0.2	0.04
Angina pectoris	678	14	2.1	340	8	2.4	0.63
Cerebral stroke/brain haemorrhage	678	8	1.2	340	4	1.3	0.80
Self-perceived Sami ethnicity	665	490	73.7	334	325	97.4	<0.001
Family history of							
Myocardial infarction	678	175	25.8	340	71	21.0	0.09
Cerebral stroke	678	165	24.3	340	88	26.0	0.57
Education≥13 years	635	172	27.1	320	132	41.2	<0.001
Ever smoking	672	418	62.2	339	202	59.6	0.43
Metabolic syndrome	675	126	18.6	338	76	22.5	0.16
Leisure-time light physical activity ≥1 hour per week	633	472	74.6	315	233	74.0	0.84
Mean age (sd)	678	53.8	(10.9)	340	51.9	(10.1)	<0.01^c^
**Minority**	**Unexposed (n=854)**			**Exposed (n=141)**			
Myocardial infarction	854	9	1.1	141	4	2.6	0.11
Angina pectoris	854	32	3.7	141	5	3.4	0.83
Cerebral stroke/brain haemorrhage	854	12	1.4	141	4	2.7	0.21
Self-perceived Sami ethnicity	826	219	26.5	141	119	84.4	<0.001
Family history of							
Myocardial infarction	854	257	30.1	141	45	32.2	0.61
Cerebral stroke	854	226	26.5	141	44	31.4	0.24
Education≥13 yrs	801	200	24.9	133	45	33.8	0.04
Ever smoking	850	569	66.9	141	103	73.0	0.16
Metabolic syndrome	849	160	18.8	141	35	24.7	0.11
Leisure-time light physical activity ≥1 hour per week	755	601	79.6	123	98	79.6	0.99
Mean age (sd)	854	53.9	(11.0)	141	51.8	(10.6)	0.04^c^

^a^Prevalence rates from logistic regression estimates.

^b^P-value from likelihood ratio tests for difference between unexposed and exposed groups.

^c^Two-sample t-test with unequal variances.

**Table 2:** **Age-adjusted characteristics**^**a **^**of the male study group by majority/minority area and marginalisation status**

	**Unexposed (n=599)**			**Exposed (n=346)**			
**Majority**	**Tot**	**n**	**%**	**Tot**	**n**	**%**	**P**^**b**^
Myocardial infarction	599	21	3.5	346	14	4.0	0.68
Angina pectoris	599	31	5.1	346	20	5.8	0.62
Cerebral stroke/brain haemorrhage	599	17	2.9	346	12	3.5	0.61
Self-perceived Sami ethnicity	589	387	65.7	342	317	92.6	<0.001
Family history of							
Myocardial infarction	599	130	21.7	346	88	25.4	0.19
Cerebral stroke	599	113	18.8	346	77	22.3	0.21
Education≥13 years	577	129	22.4	336	77	22.9	0.86
Ever smoking	596	438	73.4	342	270	78.8	0.06
Metabolic syndrome	596	61	10.3	344	45	13.2	0.18
Leisure-time light physical activity ≥1 hour per week	557	423	75.9	325	232	71.5	0.15
Mean age (sd)	599	54.5	(11.3)	346	53.7	(9.7)	0.24^c^
**Minority**	**Unexposed (n=841)**			**Exposed (n=228)**			
Myocardial infarction	841	36	4.3	228	16	6.8	0.10
Angina pectoris	841	56	6.7	228	24	10.7	0.04
Cerebral stroke/brain haemorrhage	841	22	2.6	228	10	4.4	0.14
Self-perceived Sami ethnicity	826	216	26.2	225	168	74.7	<0.001
Family history of							
Myocardial infarction	841	222	26.4	228	85	37.3	<0.01
Cerebral stroke	841	194	23.1	228	62	27.0	0.24
Education≥13 yrs	800	198	24.8	209	59	28.0	0.36
Ever smoking	837	640	76.5	224	171	76.1	0.89
Metabolic syndrome	838	92	11.0	228	26	11.5	0.84
Leisure-time light physical activity ≥1 hour per week	745	589	79.0	197	152	77.3	0.61
Mean age (sd)	841	56.1	(11.0)	228	54.2	(10.0)	0.01^c^

^a^ Prevalence rates from logistic regression estimates.

^b^ P-value from likelihood ratio tests for difference between unexposed and exposed groups.

^c^ Two-sample t-test with unequal variances.

**Table 3:** **Age-specific and total prevalence rates of self-reported lifetime cardiovascular disease**^**a **^**in men and women by majority/minority area and marginalisation status**

	**Majority**					**Minority**				
	**Unexposed**		**Exposed**			**Unexposed**		**Exposed**		
	**n**	**%**	**n**	**%**	**P**^**c**^	**n**	**%**	**n**	**%**	**P**^**c**^
**Women**	(n=678)		(n=340)			(n=854)		(n=141)		
36-49 years	2	0.8	1	0.6	0.99	1	0.3	3	4.4	0.02
50-59 years	13	6.0	6	5.6	0.99	22	8.5	4	9.5	0.77
60-79 years	35	17.9	12	16.0	0.86	57	21.8	10	32.3	0.26
Total crude	50	7.4	19	5.6	0.36	80	9.4	17	12.1	0.36
Total age-adjusted^b^	27^d^	4.0	13	3.8	0.85	44^d^	5.1	13	9.0	0.06
**Men**	(n=599)		(n=346)			(n=841)		(n=228)		
36-49 years	8	3.5	4	3.1	0.99	4	1.6	3	3.9	0.36
50-59 years	16	9.4	16	12.4	0.45	28	10.1	18	20.0	0.02
60-79 years	52	25.9	27	30.3	0.48	95	29.9	21	34.4	0.54
Total crude	76	12.7	47	13.6	0.69	127	15.1	42	18.4	0.22
Total age-adjusted^b^	59^d^	9.8	42	12.1	0.26	84^d^	10.0	36	15.8	0.02

^a^Myocardial infarction, angina pectoris, and cerebral stroke/brain haemorrhage combined.

^b^Prevalence rates from logistic regression estimates.

^c^P-value for difference between unexposed and exposed groups. Age-specific and total crude rates tested by Fisher’s exact chi-square tests. Total age-adjusted rates tested by likelihood ratio tests.

^d^Expected number of cases.

**Table 4:** **Odds ratios for self-reported lifetime cardiovascular disease**^**a **^**by selected risk factors**

	**Age-adjusted**		**Full model**^**b **^**(n=3467)**	
	**OR**	**95% CI**	**OR**	**95% CI**
Marginalisation				
Unexposed majority	1.00		1.00	
Exposed majority	1.21	0.86-1.68	1.07	0.73-1.56
Unexposed minority	1.21	0.94-1.55	1.10	0.81-1.51
Exposed minority	2.19	1.53-3.15	2.10	1.40-3.14
Self-perceived Sami ethnicity				
No	1.00		1.00	
Yes	0.93	0.76-1.14	0.88	0.66-1.17
Smoking				
Never	1.00		1.00	
Current/Previous	1.93	1.52-2.43	1.40	1.05-1.86
Leisure-time light physical activity				
< 1 hour per week	1.00		1.00	
≥ 1 hour per week	0.63	0.50-0.79	0.63	0.48-0.82
Metabolic syndrome				
No	1.00		1.00	
Yes	1.29	1.01-1.64	1.31	0.97-1.78
Family history of cardiovascular disease				
No	1.00		1.00	
Yes	1.95	1.60-2.38	1.89	1.49-2.39
Education				
0-12 years	1.00		1.00	
13+ years	0.63	0.46-0.87	0.63	0.43-0.91
Sex				
Women	1.00		1.00	
Men	1.71	1.40-2.09	1.60	1.24-2.06
Age (years)	-	-	1.11	1.09-1.12

^a^Myocardial infarction, angina pectoris, and cerebral stroke/brain haemorrhage combined.

^b^Adjusting for self-perceived Sami ethnicity, smoking, leisure-time light physical activity, metabolic syndrome, family history of cardiovascular disease, education, sex and age. All variables are mutually adjusted for each other.

All statistical analyses were performed using STATA version 12.1 (Stata Corp, College Station, TX).

## Results

Age-adjusted characteristics of the study group are presented in Tables 1 (women) and 2 (men). Among women in the Sami majority area, significant differences between unexposed and exposed in the distribution of myocardial infarction (MI) (p=0.04), self-perceived Sami ethnicity (p<0.001), education ≥13 years (p<0.001) and age (p=0.01) were observed. Among exposed women in the Sami majority area, education (41.2%) and Sami ethnicity (97.4%) were more frequent, and MI was less common (0.2%) compared with the unexposed. In the minority area, significant difference was found for self-perceived Sami ethnicity (p<0.001), education ≥13 years (p=0.04) and age (p=0.04). Sami ethnicity (84.4%) and education ≥13 years (33.8%) were more common among the exposed.

Among men in the Sami majority area, significant difference was observed only in Sami ethnicity (<0.001) which was more frequent among the exposed. In the minority area, significant difference in the distribution of angina pectoris (p=0.04), self-perceived Sami ethnicity (<0.001), family history of MI (p<0.01) and age (p=0.01) was found. Those exposed reported more angina pectoris (10.7%), Sami ethnicity (74.7%) and family history of MI (37.3%). The sensitivity analysis (see Methods) revealed no difference in CVD prevalence between the logistic regression estimates and the direct method.

Table 3 shows prevalence rates of self-reported lifetime cardiovascular disease (CVD). No difference between the exposed and unexposed groups in the total burden of CVD was found in women. Among men, a significant difference (p=0.02) was found in the minority area. In the unexposed group, 10.0% reported having ever had cardiovascular disease compared to 15.8% in the exposed group. The sensitivity analysis showed almost identical CIs in all strata, except among unexposed women in the Sami minority and majority areas; though the CI overlapped, the overlapping was less evident herein.

Table 4 presents odds ratios for CVD from age-adjusted models, and a full (mutually adjusted) model. A significant effect of exposed minority status was observed when compared to unexposed majority status (OR 2.19, 95% CI: 1.53-3.15). This effect continued after controlling for confounding and intermediate variables (OR 2.10, 95% CI: 1.40-3.14). A moderate confounding effect of leisure-time light physical activity was observed. Without the variable in the model, the OR was attenuated to 1.83 (data not shown). The sensitivity analyses (see Methods) revealed no marked differences in results. The regression was not stratified by sex, as initial analyses showed no significant sex difference (data not shown).

## Discussion

In this article, we have presented the effects of marginalisation on self-reported life-time cardiovascular disease (CVD) by Sami majority and minority areas. The main finding in this study was that marginalised Sami living in Norwegian dominated areas were more than twice as likely to report CVD as non-marginalised Sami living in Sami majority areas. A moderate confounding effect of leisure-time light physical activity was observed. We found that marginalised minority Sami were more physically active than non-marginalised majority Sami, as the OR for CVD was attenuated from 2.10 to 1.83 when the variable was removed from the model. Further analysis showed that this effect was independent of marginalisation status and largely due to a generally higher level of leisure-time light physical activity in the minority area compared with the majority area (data not shown). Leisure-time hard physical activity was not included in the models as initial analyses showed that it was insignificant (data not shown). The questionnaire did not include physical activity at work.

To our knowledge, no studies have specifically operationalized marginalisation as an independent variable relative to cardiovascular disease in indigenous populations. To a varying degree, most studies refer to marginalisation as an overarching influence; for example, Yusuf et al. [16] reported increasing prevalence of CVD among indigenous peoples in Alaska and Canada and related the burden of disease to an increase in “urban” lifestyles and the marginalised position of these peoples. Galloway [17] referred to a CVD prevalence rate of 16.4% in an American Indian/Alaska Native population, while a more recent study on ischaemic heart disease (IHD) among Greenlandic Inuit reported prevalence rates of 10.8% in men and 10.2% in women [18]. Sjölander argues that most health disparities observed in the Sami population in Sweden have their origin in the marginalisation of Sami culture, way of life and reindeer husbandry [19]. Brown et al. [20] argue that there is clearly a temporal relationship between marginalisation and the prevalence of cardiovascular disease and its conventional risk factors in indigenous populations; they further state that the conventional risk factors are unlikely to explain the high CVD burden alone. Marginalisation through socioeconomic status, loss of culture, identity and subsistence rights, and subsequent chronic stress has been suggested as driving forces of ill health in such populations [20-22]. Hansen and Sørlie [23] found that Sami males reported greater levels of stress than ethnic Norwegians and that ethnic discrimination was strongly associated with elevated levels of psychological stress; an extensive and growing body of literature acknowledges chronic stress as a causal factor in the development of ischaemic heart disease and other atherosclerotic manifestations, as well as in the development of hypertension and metabolic disturbances which fuel the atherosclerotic process [24]. However, biological stress responses do not act in isolation, but against a number of genetic, physiological and lifestyle risk factors [25].

We have included a number of risk factors such as years of education, smoking, leisure-time light physical activity and MetS. Sensitivity analyses with regard to duration and intensity of smoking, alcohol consumption and total cholesterol were also performed without observing marked effects. There are thus no obvious explanations for the excess of CVD observed among marginalised Sami in Norwegian dominated areas. We have not included any information on diet as the questionnaires did not incorporate total diet and total energy intake [5]. It is nevertheless possible that diet partly explains the relationship between marginalisation and majority/minority area, and CVD; Brustad et al. have reported geographical difference when comparing dietary patterns in areas with both Sami and Norwegian populations [26], resulting in a lower prevalence of iron deficiency in inland situated Sami areas, probably due to high intakes of high bioavailable iron from reindeer meat [27,28]. These areas roughly correspond to the majority area. Furthermore, Tynes and Haldorsen [29] found a protective effect of an assumed higher intake of reindeer meat with regard to death from diseases of the circulatory system. Nevertheless, as the regression analysis and sensitivity analysis showed little or no effect of MetS and total cholesterol, substantial intermediate effects of diet is unlikely.

Chronic stress exposure following marginalisation may however be a plausible explanation for some of the observed excess of CVD. The governmental assimilation in the period 1850-1959 was more intense in the minority areas and the reduction in agriculture and the fjord fisheries herein since World War II have influenced the economic and cultural infrastructure negatively [30]. Despite the overall strengthening of Sami language, culture and primary industries in Norway since the 1970s, the buffering effect of a growing Sami civil society today is more obvious in the majority area than in the minority area; the building of important Sami institutions inland generated and strengthened settings where Sami language and culture could become relevant and replaced to a large extent the jobs lost within the primary industry [30]. These factors may explain why marginalisation is influential in predicting CVD among Sami in Norwegian dominated areas but not among Sami in the majority areas; the stronger Sami civil society in the majority area may have a protective effect with regard to stress exposure. This exposure may well be associated with intermediate lifestyle factors which were not ascertained or included in this study. Nevertheless, previous research have documented a consistent association between chronic stress and coronary atherosclerosis (and atherosclerotic risk factors) and these relations persist after adjusting for confounding variables and lifestyle factors [31,32].

### Weaknesses

Disease status was self-reported, not medically confirmed. The reported accuracy of self-reported diagnoses is inconsistent in the literature, but population studies in northern Norway support agreement between self-reported MI and medical records [33,34]. There is however more uncertainty connected to the validity of self-reporting of stroke [33-35]; Njølstad [33] found considerable over-reporting of strokes, largely explained by the phrasing of the question to include transient ischaemic attacks. Tretli et al. [34] found poor agreement between self-reported stroke and medical records in the Finnmark population, while in Tromsø Engstad et al. [35] observed self-report to be consistent with results from clinical examinations. Utsi et al. [36] found self-reported angina and MI among Sami in Finnmark to correspond well with medical records and ECG. We have examined the relationship between self-reported angina and a two-item version of the Rose angina questionnaire. Among women reporting lifetime angina, 32% reported having symptoms consistent with angina. Only 4% of those without a history of angina reported having such symptoms. In men, the proportions were 35% and 7%, respectively (data not shown). This may provide some support to the validity of the self-reported data. In light of this, and results from previous studies using a comparable formulation of questions in similar populations [34-36], we believe that our estimates are valid, given that our ambition was to identify individuals who have ever experienced angina, MI and/or cerebral stroke/brain haemorrhage.

We have limited information about non-responders other than that they tended to be young, single and male; we do not know the Sami participation rate as the information on ethnicity was ascertained by self-report [4]. The participation rate of 61% could have introduced selection bias. It is difficult to assess the direction of the selection bias while we lack information about the non-participants’ risks factor pattern and CVD status [4]. Nevertheless, differences between responders and non-responder are often important but seldom so great that studies are irrevocably undermined [37].

### Strengths

A relatively good response rate (61%) and a fairly large sample have enabled a comparative analysis within the Sami population. With the exception of Nordland county, the sample is representative for the general rural Sami population in the region [4]. Such an in-depth analysis of CVD burden within the Sami population has never been done with regard to marginalisation and minority/majority status. This study also targets sociocultural systems within the Sami population as a unit of analysis and thus adds to the knowledge of the social determinants of health in the Sami population in Norway.

## Conclusions

This study showed that marginalised Sami living in Norwegian dominated areas were more than twice as likely as non-marginalised Sami from Sami majority areas to report lifetime cardiovascular disease (CVD). Moderate to no intermediate effects were seen after including established CVD risk factors, which suggest little difference in lifestyle related factors. Chronic stress exposure following marginalisation may however be a plausible explanation for some of the observed excess of CVD. Our cross-sectional study design does not however allow any conclusion with regard to causality and risk.

## Abbreviations

WWII: World War II; SAMINOR: Population-based study of health and living conditions in areas with both Sami and Norwegian populations; CVD: Self-reported lifetime cardiovascular disease; NIPH: Norwegian institute of public health; MI: Myocardial infarction; WHO: World health organization; WC: Waist circumference; STATA: Data analysis and statistical software; OR: Odds ratio; 95% CI: 95% confidence interval.

## Competing interests

The authors declare that they have no competing interests.

## Authors’ contributions

BME performed the statistical analysis and drafted the manuscript. MM, KLH and ARB helped to draft the manuscript, and MM also assisted with the statistical analysis. All authors read and approved the final manuscript.

## Pre-publication history

The pre-publication history for this paper can be accessed here:

http://www.biomedcentral.com/1471-2458/13/522/prepub

## Supplementary Material

Additional file 1 Initial and main questionnaires. Click here for file

Additional file 2 Additional questionnaire. Click here for file
