# Supplementary material for: Marginalisation and cardiovascular disease among rural Sami in Northern Norway: a population-based cross-sectional study
Source: BMC Public Health. 2013 May 29;13:522. doi: 10.1186/1471-2458-13-522 (PMC3668238; doi:10.1186/1471-2458-13-522)
Supplement: Additional file 1 — Initial and main questionnaires. [file 1471-2458-13-522-S1.pdf]

1. YOUR OWN HEALTH

What is your current state of health? *(Mark only one)*

☐ Poor ☐ Not so good ☐ Good ☐ Very good

**Do you have or have you had?**	Yes	No	Age first time
Asthma	☐	☐	
Chronic bronchitis, emphysema, COPD	☐	☐	
Diabetes	☐	☐	
Fibromyalgia/chronic pain syndrome	☐	☐	
Psychological problems for which you have sought help	☐	☐	
Myocardial infarction (heart attack)	☐	☐	
Angina pectoris (heart cramp)	☐	☐	
Cerebral stroke/brain haemorrhage	☐	☐	
Multiple sclerosis	☐	☐	
Ulcerous colitis	☐	☐	

	Yes	No
**Do you get pain or discomfort in the chest when walking up hills or stairs, or walking fast on level ground?**	☐	☐
**Do you get such pain or discomfort even if you are resting?**	☐	☐

2. MUSCULO AND SKELETAL PAIN

	Yes	No
**Have you** *during the last year* **suffered from pain and/or stiffness in muscles or joints that has lasted for** *at least 3 months*?	☐	☐

**Have you ever had:**	Yes	No	Age last time
**a wrist/forearm fracture?**	☐	☐	
**a hip fracture?**	☐	☐	

3. STOMACH AND INTESTINAL SYMPTOMS

	Yes	No
**Have you experienced pyrosis/heartburn almost daily for at least a week?**	☐	☐
**Have you ever had pains/aches in the stomach lasting for at least 2 weeks?**	☐	☐

If yes, where in the stomach are the pains situated? *(Mark only one)*

☐ Upper part ☐ Lower part ☐ The whole stomach

Normally, for how long time are the stomach pains present?
(Mark one)

For periods of weeks length	☐
For periods of months length	☐
Always	☐

	Yes	No
**Do you often suffer from flatulence, rumbling in the stomach or much wind?**	☐	☐

Is your stool usually:

☐ Normal ☐ Loose ☐ Hard and lumpy
☐ Alternating hard and loose ☐ Smelly

	Yes	No
**Do you for some periods of time have three stools per day or more?**	☐	☐
**Have you had stomach/intestinal problems after consuming milk?**	☐	☐

Are there others in your family with similar stomach symptoms?

☐ Mother ☐ Father ☐ Siblings ☐ Child ☐ None

4. OTHER PAINS/PROBLEMS

Listed below are some symptoms or problems. Have you experienced any of these *during the last week* **(including today)?**
(Tick one box for each item)

	Not affected	Slightly affected	Affected quite a lot	Severely affected
Sudden unfounded fears	☐	☐	☐	☐
Felt frightened or anxious	☐	☐	☐	☐
Faintness or dizziness	☐	☐	☐	☐
Felt tense or upset	☐	☐	☐	☐
Had a tendency to easily blame yourself	☐	☐	☐	☐
Insomnia/sleeplessness	☐	☐	☐	☐
Felt dejected or melancholic	☐	☐	☐	☐
Had a feeling of being useless/of little value	☐	☐	☐	☐
Felt that everything is a struggle	☐	☐	☐	☐
Feeling of hopelessness regarding the future	☐	☐	☐	☐
Had thoughts of ending your life	☐	☐	☐	☐

5. ILLNESS IN THE FAMILY

	Yes	No	Don't know
**Have one or more of your parents or siblings had a heart attack or angina (heart cramp)?**	☐	☐	☐

Tick off relatives who have, or have ever had, any of the following conditions, and report the age of when they got the illnesses.
(If several siblings, report the one who got the illness at the youngest age)

	Mother	Father	Sister	Brother	Child	None	Age first time
Myocardial infarction before age 60	☐	☐	☐	☐	☐	☐	
Myocardial infarction after age 60	☐	☐	☐	☐		☐	
Diabetes	☐	☐	☐	☐	☐	☐	
Cerebral stroke or brain haemorrhage	☐	☐	☐	☐	☐	☐	
Asthma	☐	☐	☐	☐	☐	☐	
Colon cancer	☐	☐	☐	☐	☐	☐	
Breast cancer	☐		☐		☐	☐	
Ovarian cancer	☐		☐		☐	☐	

	Brothers	Sisters
**How many siblings do you have?**		

6. USE OF MEDICATION

Medicines, in this context, means medicines bought at a pharmacy. Food supplements and vitamins are not included here.

**Do you take?**	Currently	Previously, but not now	Never used
Medications for high blood pressure	☐	☐	☐
Cholesterol reducing medication	☐	☐	☐
Insulin	☐	☐	☐
Tablets for diabetes	☐	☐	☐

How often during the last 4 weeks have you used the following medications? *(Tick one box for each line)*

	Not used for the last 4 weeks	Less frequently than every week	Every week, but not daily	Daily
Pain killers without prescription	☐	☐	☐	☐
Pain killers with prescription	☐	☐	☐	☐
Sleeping pills	☐	☐	☐	☐
Tranquilizers	☐	☐	☐	☐
Antidepressants	☐	☐	☐	☐
Other prescribed medicines	☐	☐	☐	☐

For those medicines you have ticked off in the last two items, and you have taken *during the last 4 weeks*:
State the name of the medicines and your reason for taking/ having taken them (disease, sympton): *(Tick one box for each line)*

Brand name of medicine *(one name per line)*	Reason for use of medicine	For how long time? Up to one year	One year or more
		☐	☐
		☐	☐
		☐	☐
		☐	☐
		☐	☐

If there is not enough space here, continue on a separate page and enclose it with the form.

7. FOOD AND BEVERAGES

How often do you usually eat the following foods?

	Rarely/ never	1–3 t. p. month	1–3 t. p. week	4–6 t. p. week	1–2 t. p. day	3 t. or more p. day
Fruit	☐	☐	☐	☐	☐	☐
Berries	☐	☐	☐	☐	☐	☐
Cheese (all types)	☐	☐	☐	☐	☐	☐
Potatoes	☐	☐	☐	☐	☐	☐
Boiled vegetables	☐	☐	☐	☐	☐	☐
Fresh vegetables/salad	☐	☐	☐	☐	☐	☐

What type of fat do you usually use? *(Tick one box for each line)*

	Do not use	Butter	Hard margarine	Soft/light margarine	Oils	Other
On bread	☐	☐	☐	☐	☐	☐
For cooking	☐	☐	☐	☐	☐	☐

Do you use the following food supplements?

	Yes, daily	Sometimes	No
Cod liver oil or cod liver oil capsules	☐	☐	☐
Fish oil capsules (omega 3)	☐	☐	☐
Vitamins and/or mineral supplement	☐	☐	☐

How much do you normally drink of the following?
(Tick one box for each line)

	Rarely/ never	1–6 glasses per week	1 glass per day	2–3 glasses per day	4 glasses a day or more
Full milk, full-fat curdled milk and yoghurt	☐	☐	☐	☐	☐
Semi-skimmed milk, semi-skimmed curdled milk and low-fat yoghurt	☐	☐	☐	☐	☐
Skimmed milk and skimmed curdled milk	☐	☐	☐	☐	☐
Semi-skimmed milk	☐	☐	☐	☐	☐
Fruitjuice	☐	☐	☐	☐	☐
Water	☐	☐	☐	☐	☐
Soft-drinks/cola-drinks with sugar	☐	☐	☐	☐	☐
Soft-drinks/cola-drinks without sugar	☐	☐	☐	☐	☐

How many cups of coffee and tea do you usually drink per day?
(Write 0 for the types you do not drink daily)

	Number of cups
Filtered coffe	☐☐
Boiled coffee (coarsely ground coffee for brewing)	☐☐
Other coffee	☐☐
Tea	☐☐

How often during the last year have you consumed alcohol?
(Low alcohol beer and non-alcoholic beer are not included)

- Never consumed alcohol ☐
- Not during the last year ☐
- A few times during the last year ☐
- 1 time per month ☐
- 2–3 times per month ☐
- 1 time per week ☐
- 2–3 times per week ☐
- 4–7 times per week ☐

To those who have consumed alcohol during the past year:
When you drink alcohol, how many glasses or drinks do you normally drink? Antall ☐☐
Approximately how many times during the last year have you consumed alcohol equivalent to 5 glasses or drinks within 24 hours? Antall ganger ☐☐

When you drink alcohol, do you normally drink:
(Tick one or more boxes)
☐ Beer ☐ Wine ☐ Spirits

8. SMOKING AND SNUFF USE

How many hours a day do you normally spend in smoke-filled rooms? *Number of whole hours* ☐☐

Did any of the adults smoke at home while you were growing up? Yes ☐ No ☐

Do you currently, or did you previously live together with a daily smoker after your 20th birthday? . . . . ☐ ☐

**Are you currently, or were you preciously a daily smoker?**	Yes, currently	Yes, previously	Never
	☐	☐	☐

**If current daily smoker, do you smoke**	Yes	No
Sigarettes	☐	☐
Cigars/cigarillos/pipe	☐	☐
Rolling tobacco	☐	☐

If you previously smoked daily, how many years is it since you stopped smoking? . . . . *Number of years* ☐☐

If you currently smoke, or have smoked before, how many cigarettes do/did you smoke per day? . . . . . *Number of cigarettes* ☐☐

If you currently smoke, or have smoked before, how old were you when you began smoking daily? *Age in years* ☐☐

If you currently smoke, or have smoked before, how many years in all have you smoked daily? *Number of years* ☐☐

**Do you take or have you been taking snuff daily?**	Yes, currently	Yes, previously	Never
	☐	☐	☐

If you have been taking snuff, for how many years in all have you been taking snuff? . . . . *Number of years* ☐☐

9. EXERCISE AND PHYSICAL ACTIVITY

How has your physical activity *in leisure time* **been** *during this last year*? *(Think of your weekly average for the year. Time spent going to work count as leisure time. Answer both questions)*

	Hours per week			
	None	Less than 1 hour	1–2 hours	3 hours or more
**Light activity** (not sweating or out of breath)	☐	☐	☐	☐
**Hard physical activity** (sweating/out of breath)	☐	☐	☐	☐

Describe your exercise and physical exertion *in leisure time*. **If your activity varies much, for example between summer and winter, then give an average. The question refers** *only to the last twelve months*. *(Tick the box that is most appropriate)*

Reading, watching TV, or other sedentary activity . . . . ☐

Walking, cycling, or other forms of exercise *at least 4 hours a week* *(This should include walking or cycling to work, Sunday stroll/walk, etc.)* ☐

Participation in recreational sports, heavy gardening, etc. *(note: duration of activity at least 4 hours a week)* . . . . ☐

Participation in hard training or sports competitions regularly and *several times a week* . . . . ☐

10. EDUCATION AND WORK

How many years of schooling/education have you completed? *(Count all years you have attended school or been studying)* . . . . *Number of years* ☐☐

How content are you with your job?
☐ Very content ☐ Content ☐ Discontent ☐ Very discontent

**Do you believe that you are in danger of losing your current work or income within the next 2 years?**	Yes	No
	☐	☐

**Do you receive any of the following benefits?**	Yes	No
Sickness benefit/Sick pay	☐	☐
Rehabilitation benefit	☐	☐
Social welfare benefits	☐	☐
Transition benefit for single parents	☐	☐

11. THE REST OF THE QUESTIONNAIRE IS TO BE ANSWERED BY WOMEN ONLY

How old were you when you started menstruating? . . . . Age in years ☐☐

If you no longer menstruate, how old were you when you stopped menstruating? . . . . Age in years ☐☐

Are you pregnant at the moment?
☐ Yes ☐ No ☐ Uncertain ☐ Above fertile age

How many children have you given birth to? . . . . *Number of children* ☐☐

If you have given birth, enter what year each child was born and how many months you did breastfeed after the birth?
(If you didn't breastfeed, write 0)

Children	Year of birth	Breastfed number of months
1. child	☐☐☐☐	☐☐
2. child	☐☐☐☐	☐☐
3. child	☐☐☐☐	☐☐
4. child	☐☐☐☐	☐☐
5. child	☐☐☐☐	☐☐

(If more children, use an extra sheet of paper)

Do you use or have you ever used? *(Tick one box for each line)*

	Currently	Previously, but not now	Never used
Contraceptive pills/minipill/ contraceptive injection?	☐	☐	☐
Hormonal intrauterine device?	☐	☐	☐
Estrogen (tablets or patches)?	☐	☐	☐
Estrogen (cream or suppositories)?	☐	☐	☐

If you use/have used prescription obliged estrogen, for how many years have you used it? . . . *Number of years* ☐☐

If you use contraceptive pills, hormonal intrauterine device, or estrogen, what brand do you currently use? *Specify*

☐

USE OF HEALTH SERVICES

How many times *during the past year* **have you personally used?**
(Tick one box for each line)

	None	1–3 times	4 +
GP (general practitioner)	☐	☐	☐
Medical specialist	☐	☐	☐
Emergency GP	☐	☐	☐
Admission to a hospital	☐	☐	☐
Home nursing care	☐	☐	☐

	None	1–3 times	4+
Home aid, organised by the municipality	☐	☐	☐
Physiotherapist	☐	☐	☐
Chiropractor	☐	☐	☐
Dentist	☐	☐	☐
Alternative medical practitioner	☐	☐	☐

How many doctors have you been seeing for the last 12 months? *(Number)* ☐☐

Have you been given a regular GP, whose name you know? Yes ☐ No ☐

When you are being examined, which language do you and your doctor communicate in? *(Tick one or more boxes)*
☐ Norwegian ☐ Sami ☐ Use an interpreter
☐ Other language

Do you think it happens that you and your doctor misunderstand each other due to linguistic problems?
☐ Never ☐ Rarely ☐ Sometimes ☐ Often ☐ Not sure

If an interpreter is needed, is your doctor good enough to request it?
☐ Yes, always ☐ Yes, most of the time ☐ No, not always
☐ No, never ☐ Don't like to use interpreter

How satisfied/dissatisfied are you with the following aspects with the municipal health service in your municipality?
(Tick one box for each line)

	Very satisfied	Satisfied	Dis-satisfied	Don't know
The distance to your doctor?	☐	☐	☐	☐
Your doctor's availability on telephone?	☐	☐	☐	☐
How soon you can get an appointment with your doctor?	☐	☐	☐	☐
How long time you are allowed with your doctor?	☐	☐	☐	☐
Your possibility to explain about you pains and problems?	☐	☐	☐	☐
Your doctor's understanding of your cultural background?	☐	☐	☐	☐
The information your doctor gives about your health and the examination and treatment you get?	☐	☐	☐	☐
Your doctor's language skills (Sami or Norwegian)?	☐	☐	☐	☐
The local health services in your municipality totally?	☐	☐	☐	☐
On the whole, how satisfied/ dissatisfied are you with the local health services in your municipality?	☐	☐	☐	☐

How long is it since you last went to see a doctor? *(Report whole numbers)* Years ☐☐ Months ☐☐

If you have ever used an alternative practitioner, which did you use? *(Tick one or more boxes)*
A traditional healer *(guvllar, reader, "blåser", laying on of hands)* ☐
A (modern) healer ☐
An acupuncture practitioner ☐
A zone therapist, homeopath, kinesiologist etc. ☐

How long is it since you last used an alternative practitioner? *(Report whole numbers)* Years ☐☐ Months ☐☐

Suppose that you would get the need for help/assistance from the local health- and social services *(home nursing care, home assistance services, social services, physiotherapy etc.)***.**

	Yes	No	Uncertain
Do you know where to approach?	☐	☐	☐
Do you feel confident that you will receive help if you need it?	☐	☐	☐
If you today receive help from the local health and social services, are you satisfied with the help they offer?	☐	☐	☐

INJURIES/ACCIDENTS

Have you been in accidents that resulted in treatment by a doctor and/or hospital admission?

	Yes	No	Number of times
Doctor	☐	☐	☐☐
Hospital admission	☐	☐	☐☐

If yes, what kind of accidents have you been treated for?

	At work	At home	During leisure time	No
Car accident	☐	☐	☐	☐
Motor cycle accident	☐	☐	☐	☐
Snowmobile accident	☐	☐	☐	☐
4-wheel motor cycle	☐	☐	☐	☐
Tractor	☐	☐	☐	☐
Accident by falling	☐	☐	☐	☐
Cutting injury	☐	☐	☐	☐
Other	☐	☐	☐	☐

Has/have the accident(s) lead to reduced ability to work?
☐ Completely ☐ Partly ☐ Not at all

FAMILY AND LINGUISTIC BACKGROUND

In Northern Norway there live people of different ethnic background. That is, they speak different languages and have different cultures. Examples of ethnic background, or ethnic group, is Norwegian, Sami and Kven.

Which language did/do you, your parents and grand parents speak at home? *(Tick one or more boxes)*

	Nor-weg-ian	Sami	Kven	Other, specify
Mother's father	☐	☐	☐	☐
Mother's mother	☐	☐	☐	☐
Father's father	☐	☐	☐	☐
Father's mother	☐	☐	☐	☐

	Nor-weg-ian	Sami	Kven	Other, specify	
Father	☐	☐	☐	☐	
Mother	☐	☐	☐	☐	
Myself.	☐	☐	☐	☐	

What is your, your father's, and your mother's ethnic background? *(Tick one or more boxes)*

	Nor-weg-ian	Sami	Kven	Other, specify	
My ethnic background . . .	☐	☐	☐	☐	
My ethnic background . . .	☐	☐	☐	☐	
My ethnic background . . .	☐	☐	☐	☐	

What do you consider yourself to be? *(Tick one or more boxes)*
☐ Norwegian ☐ Sami ☐ Kven
☐ Other, specify:

EMPLOYMENT/ECONOMY

What type of work/livelihood do you have? *(Tick one or more boxes)*
☐ Full time job with a fixed salary
☐ Part time job with a fixed salary
☐ Seasonal work ☐ Self-employed
☐ Unemployed ☐ Homemaker (fulltime housework)
☐ Old-age pension ☐ Disability pension
☐ Other, specify:

Would you be willing to move if you were offered work somewhere else?
☐ Yes ☐ No ☐ Parts of the year ☐ Uncertain

If you are out of work, for how long have you been seeking employment? *(Report whole numbers)* Years Months

If you are self-employed, what do you work with?
(Tick one or more boxes)
☐ Reindeer herding? ☐ Fishing? ☐ Farming?
☐ Forestry? ☐ Business?
☐ Other, specify:

How many persons are living in your household?. . . . . *(Number of persons)*

How large is your family's/household's gross income each year?
☐ Less than 150 000 NOK ☐ 150 000–300 000 NOK
☐ 301 000–450 000 NOK ☐ 451 000–600 000 NOK
☐ 601 000–750 000 NOK ☐ More than 750 000 NOK

How often do you participate in gambling (national lottery, football betting, gambling machines etc.)?
☐ Never/rarely ☐ 1–3 times a month ☐ Once a week
☐ 2–6 times a week ☐ Daily

For how much money do you on average gamble per week?
☐ Less than 100 NOK ☐ 100–500 NOK
☐ 501–1000 NOK ☐ More than 1000 NOK

BULLYING

By bullying we mean when one or more persons *systematically and over time* **say or do bad things against you, and you have difficulty in defending yourself against them.**

Have you experienced bullying?
☐ Yes, for the last 12 months ☐ Yes, previously ☐ No

If you have been bullied, what kind of bullying did you experience?
(Tick one or more boxes)
☐ Talking behind your back/gossip ☐ Being ignored
☐ Discriminating remarks
☐ Other, specify:

Can you state where the bullying takes/took place?
☐ At school ☐ At boarding school/dormitory
☐ At work ☐ In local community
☐ Other, specify:
