# Supplementary material for: Marginalisation and cardiovascular disease among rural Sami in Northern Norway: a population-based cross-sectional study
Source: BMC Public Health. 2013 May 29;13:522. doi: 10.1186/1471-2458-13-522 (PMC3668238; doi:10.1186/1471-2458-13-522)
Supplement: Additional file 2 — Additional questionnaire. [file 1471-2458-13-522-S2.pdf]

ADDITIONAL QUESTIONS TO THE HEALTH- AND LIVING CONDITIONS SURVEY

Thank you for attending our survey of health- and living conditions. By doing so, you have contributed to new knowledge about health and living conditions in areas with Sámi and Norwegian settlements. The main aim of the survey has been to obtain new knowledge about cardiovascular diseases in order to prevent them. Additionally, the survey shall bring us knowledge about other diseases and pains in order to get an overview of people's health in your county. We therefore ask you to answer some questions about things that may have impact on these and other diseases.

The completed questionnaire is to be posted in the enclosed envelope. Postage is paid. Thank you in advance for your help!

Best regards, Centre for Sámi Health Research and National Institute of Public Health.

Date for filling in questionnaire: Day ☐☐ Month ☐☐ Year ☐☐

1. SYMPTOMS

	Yes	No
**Do you cough about daily for some periods of the year?**	☐	☐
Have you had such cough for as long as a 3 month period during both of the last two years?	☐	☐

	Yes	No
**Do you from time to time suffer from sleeplessness/ insomnia?**	☐	☐

If yes, when do you suffer from it the most? *(Tick one or more boxes)*
☐ All seasons ☐ Spring ☐ Summer ☐ Autumn ☐ Winter

	Yes	No
Have you in the last twelve months suffered from sleeplessness to the extent that it has affected your ability to work?	☐	☐

On the whole, are you content with your way of life?
☐ Very content ☐ Quite content ☐ Discontent
☐ Very discontent

	Yes	No
Does it happen that you for longer periods (>14 days) feel sad and depressed?.	☐	☐

During the last 14 days, have you felt unable to cope with your difficulties?
☐ No ☐ Sometimes ☐ Often ☐ Almost all the time

Do you sometimes feel lonely?
☐ No ☐ Sometimes ☐ Often

2. CURRENT DIET / FOOD HABITS

We would like to ask you some questions about how often you eat different food items. Think of an average over the last year. Tick one box per line for number of times. If you do not remember exactly, fill in the best you can.

How many times per week do you usually eat dinner? . . . . *(Number of times)* ☐☐

How often does your dinner include:

	Never/ rarely	Per month 1	Per month 2–3	Per week 1	Per week 2	Per week 3	Per week 4	Per week 5+
Fish	☐	☐	☐	☐	☐	☐	☐	☐
Meat (whole, ground). . . .	☐	☐	☐	☐	☐	☐	☐	☐
Not fish or meat	☐	☐	☐	☐	☐	☐	☐	☐

How often do you eat *poached* cod or coalsfish for dinner?

	Never	1–11 per year	Per month 1	Per month 2–3	Per week 1	Per week 2	Per week 3+
Cod (eg. fresh, cured, smoked, dried)	☐	☐	☐	☐	☐	☐	☐
Coalfish (eg. fresh, dried).	☐	☐	☐	☐	☐	☐	☐

How often do you eat other poached fish for dinner?

	Never	Per year 1–5	Per year 6–11	Per month 1	Per month 2–3	Per week 1	Per week 2+
Fatty fish (eg. poached salmon, halibut, ocean perch, char, trout, powan)	☐	☐	☐	☐	☐	☐	☐
Lean fish (e.g. poached haddock, perch, pike, grayling).	☐	☐	☐	☐	☐	☐	☐

How often do you eat fried fish for dinner?

	Never	Per year 1–5	Per year 6–11	Per month 1	Per month 2–3	Per week 1	Per week 2+
Fatty fish (eg. fried salmon, halibut, ocean perch, char, trout, powan)	☐	☐	☐	☐	☐	☐	☐
Lean fish (e.g. fried haddock, perch, pike, grayling)	☐	☐	☐	☐	☐	☐	☐

How often do you eat processed fish for dinner?

	Never	Per year 1–5	Per year 6–11	Per month 1	Per month 2–3	Per week 1	Per week 2+
Fish cakes/balls/pudding	☐	☐	☐	☐	☐	☐	☐
Fish au gratin or fish in white sauce	☐	☐	☐	☐	☐	☐	☐
Fish fingers/breaded fish	☐	☐	☐	☐	☐	☐	☐

How often do you eat fish on bread:

	Never	1–11 per year	Per month 1	Per month 2–3	Per week 1–2	Per week 3–4	Per week 5+
Cured/salted fish	☐	☐	☐	☐	☐	☐	☐
Smoked fish	☐	☐	☐	☐	☐	☐	☐
Mackerel in tomato sauce. . .	☐	☐	☐	☐	☐	☐	☐
Pickled herring	☐	☐	☐	☐	☐	☐	☐
Smoked cod caviar	☐	☐	☐	☐	☐	☐	☐
Other fish spreads	☐	☐	☐	☐	☐	☐	☐

How many times per year do you eat fish liver and hard roe?

	0	1-3	4-6	7-9	10+
Fish liver	☐	☐	☐	☐	☐
Fish roe	☐	☐	☐	☐	☐

How often do you eat the following dishes:

	Never	Per year 1–5	Per year 6–11	Per month 1	Per month 2–3	Per week 1	Per week 2+
Pizza.	☐	☐	☐	☐	☐	☐	☐
Spaghetti, pasta dishes.	☐	☐	☐	☐	☐	☐	☐
Hamburgers in a bun	☐	☐	☐	☐	☐	☐	☐
Meatballs/hamburger patties	☐	☐	☐	☐	☐	☐	☐
Sausages	☐	☐	☐	☐	☐	☐	☐
Stew/casserole.	☐	☐	☐	☐	☐	☐	☐

Skjema 2.indd 1 19-02-08 12:53:23

How often do you eat pure meat for dinner?

(eg. chops, roast, casserole, steak, filet)

		Per year		Per month		Per week	
	Never	1–5	6–11	1	2–3	1	2+
Chicken	☐	☐	☐	☐	☐	☐	☐
Pork	☐	☐	☐	☐	☐	☐	☐
Beef	☐	☐	☐	☐	☐	☐	☐
Mutton/lamb	☐	☐	☐	☐	☐	☐	☐
Elk meat	☐	☐	☐	☐	☐	☐	☐
Whale meat	☐	☐	☐	☐	☐	☐	☐

	Number of eggs				
	0	1-3	4-6	7-9	10+
**How many eggs from sea birds do you eat per year?**	☐	☐	☐	☐	☐

**How often do you eat reindeer meat?**	Never	1–11 per year	Per month 1	2–3	Per week 1	2	3+
Boiled reindeer meat (bouillon)	☐	☐	☐	☐	☐	☐	☐
Fried reindeer meat (whole, scraped, minced)	☐	☐	☐	☐	☐	☐	☐
Smoked reindeer meat	☐	☐	☐	☐	☐	☐	☐
Dried reindeer meat	☐	☐	☐	☐	☐	☐	☐

**How often do you eat other reindeer products?**	Never	Per year 1–5	6–11	Per month 1	2–3	1+ per week
Dishes with reindeer blood	☐	☐	☐	☐	☐	☐
Marrow bone	☐	☐	☐	☐	☐	☐
Reindeer tongue	☐	☐	☐	☐	☐	☐
Reindeer liver	☐	☐	☐	☐	☐	☐

How often do you eat berries?

One time corresponds to jam on one slice of bread, wild cranberries to one serving of dinner, one portion of desert, 1 glas of syrup, or a trip where you ate wild berries.

		Per year		Per month		Per week	
**Cloudberries**	Never	1–5	6–11	1	2–3	1–2	3+
Fresh, frozen, stirred	☐	☐	☐	☐	☐	☐	☐
Cooked/purchased jam	☐	☐	☐	☐	☐	☐	☐
**Cranberries**	Never	1–5	6–11	1	2–3	1–2	3+
Fresh, frozen, stirred	☐	☐	☐	☐	☐	☐	☐
Cooked/purchased jam	☐	☐	☐	☐	☐	☐	☐
**Bilberries**	Never	1–5	6–11	1	2–3	1–2	3+
Fresh, frozen, stirred	☐	☐	☐	☐	☐	☐	☐
Cooked/purchased jam	☐	☐	☐	☐	☐	☐	☐
Syrup	☐	☐	☐	☐	☐	☐	☐
**Crowberries**	Never	1–5	6–11	1	2–3	1–2	3+
Fresh, frozen, stirred	☐	☐	☐	☐	☐	☐	☐
Syrup	☐	☐	☐	☐	☐	☐	☐

How does your household usually acquire/get hold of the following foods for private use? *(Tick one or more boxes)*

	Never/ rarely eaten	Self sufficient: Completely	Partly	Buy it in/from: Shops	Private salesman	Get it for free or in exchange
**Meat**						
Reindeer	☐	☐	☐	☐	☐	☐
Mutton	☐	☐	☐	☐	☐	☐
Elk	☐	☐	☐	☐	☐	☐
**Fish**						
Fresh water fish	☐	☐	☐	☐	☐	☐
Sea fish	☐	☐	☐	☐	☐	☐
**Berries**						
Cloudberries	☐	☐	☐	☐	☐	☐
Wild cranberries	☐	☐	☐	☐	☐	☐

**How often do you hunt, fish or pick berries?**	Never	Rarely	Sometimes	Much of my leisure time
Hunt for ptarmigan/small game	☐	☐	☐	☐
Hunt for big game	☐	☐	☐	☐
Go fishing	☐	☐	☐	☐
Pick berries	☐	☐	☐	☐

**How often did you eat a main meal from your household's hunting/fishing in the last year?**	Never	Per year 1–5	6–11	Per month 1	2–3	Per week 1	2+
Hunting	☐	☐	☐	☐	☐	☐	☐
Fishing	☐	☐	☐	☐	☐	☐	☐

3. DIET DURING CHILDHOOD/ADOLESCENCE

Think of the food you got at home before you moved out. If you stayed most of the time at boarding school, think of the food you got there.

Did you attend boarding school (public or private) in primary school (grade 1–6) or lower secondary school (grade 7–9)?

☐ Yes, in lower secondary school ☐ Yes, in primary school

☐ Yes, both in primary and lower secondary school ☐ Neither

If yes, for how many grades did you attend boarding school? ☐☐

For how many months were you at boarding school, on average, each grade?

1–3	4–6	7–9
☐☐	☐☐	☐☐

**How often did you eat fish and reindeer meat in you childhood?**	Never	1–11 per year	Per month 1	2–3	Per week 1–2	3–4	5+
Boiled/fried fish	☐	☐	☐	☐	☐	☐	☐
Reindeer meat	☐	☐	☐	☐	☐	☐	☐

**How often did you eat the following in your childhood:**	Never	1–11 per year	Per month 1	2–3	Per week 1	2	3+
Blood products	☐	☐	☐	☐	☐	☐	☐
Sheep meat (mutton/lamb)	☐	☐	☐	☐	☐	☐	☐
Meatballs and sausages	☐	☐	☐	☐	☐	☐	☐
Fish products	☐	☐	☐	☐	☐	☐	☐
Fish liver and roe	☐	☐	☐	☐	☐	☐	☐
Porridge and pancakes	☐	☐	☐	☐	☐	☐	☐

Skjema 2.indd 2 19-02-08 12:53:24

	Yes	No
Were you given medical cod liver oil during childhood?	☐	☐
Were you served cod liver oil with e.g. fish (instead of other fat)?	☐	☐

**How often did you eat wild berries and plants in your childhood:**	Never	Per year 1–5	Per year 6–11	Per month 1	Per month 2–3	Per week 1–2	Per week 3+
Wild berries	☐	☐	☐	☐	☐	☐	☐
Mountain sorrel (Oxyria digyna)	☐	☐	☐	☐	☐	☐	☐
Angelica (Angelica archangelica)	☐	☐	☐	☐	☐	☐	☐

Is the food you eat today different from what you were served during your childhood?
☐ No ☐ Slightly different ☐ Quite different ☐ Very different

4. EATING AT NIGHT

	Yes	No
**Do you often wake up during the night to eat?**	☐	☐

If yes, answer the following 4 questions:

When do you most often suffer from this problem? *(Tick one or more boxes)*
☐ All year ☐ Spring ☐ Summer
☐ Autumn ☐ Winter

What do you eat at night?
☐ Meat ☐ Bread/sandwiches ☐ Candy
☐ Other

	Yes	No	Don't know
**During a 24 hour period, do you eat more than half the food amount after 8 o'clock in the evening?**	☐	☐	
**Do other members of your family suffer from eating at night?**	☐	☐	☐
**Do you work shift, at night or are you at call?**	☐	☐	

5. FAMILY AND FRIENDS

In which municipalities have you lived for at least a year?

	Municipality	Years From	Years To
1.	(Birthplace)	0	
2.			
3.			
4.			
5.			

(If you have lived in more municipalities, use a separate sheet of paper)

	Yes	No
Do you live with a spouse/partner?	☐	☐
Do you have shared or daily care for Children?	☐	☐
Parents/others?	☐	☐

How many good friends do you have? *(With whom you can talk confidentially and who can give you help when you need it? Do not count people you live with.)* *(Number of friends)* ☐☐

Are you connected to/member of any of these churches/religious communities? *(Tick one or more boxes)*
☐ Member of the State church ☐ The Laestadian community
☐ Other church/religious community ☐ Not member of any churc/community

Do you feel you have influence on what is happening in you local community? *(Tick one box only)*
☐ Yes, to a large extent ☐ Yes, to some extent
☐ Yes, to a small extent ☐ No ☐ Have not tried

6. PERSONAL VALUES

To be answered by all:

	Very important	Important	Less important	Insignificant
Is it important to you to have contact with nature?	☐	☐	☐	☐
Is exploitation of nature through fishing, hunting and berry-picking important to you?	☐	☐	☐	☐
Is maintenance of family traditions important to you?	☐	☐	☐	☐

Have you experienced bullying/discrimination due to your ethnical (Sámi, Kven, Russian, Tamil, Norwegian etc.) background?
☐ Many times ☐ Sometimes ☐ Rarely ☐ Never

Do you think discrimination of ethnic minorities can have negative impact on health?
☐ To a large extent ☐ To some extent ☐ To a small extent
☐ Absolutely not

Do you feel you are being forced from you work/trade?
☐ To a large extent ☐ To some extent ☐ To a small extent
☐ Absolutely not

7. QUESTIONS TO THOSE WITH SÁMI BACKGROUND

	Very important	Important	Less important	Insignificant
Are Sámi clothing traditions important to you?	☐	☐	☐	☐
How important is Duodji to you?	☐	☐	☐	☐
What does maintenance and development of the Sami language mean to you?	☐	☐	☐	☐
Is it important to you to live in a community where you can meet other Sami on a daily basis?	☐	☐	☐	☐
Do you think maintenance of typical Sami industries is important?	☐	☐	☐	☐
Is development of the modern Sami school system important to you?	☐	☐	☐	☐
Is it important with more modern work places in Sami communities?	☐	☐	☐	☐
What does Sámi media (radio, TV, newspapers, books) mean to you?	☐	☐	☐	☐
What does modern Sámi art mean to you?	☐	☐	☐	☐
What do you think of the stronger international contact the Sámi society and culture have obtained?	☐	☐	☐	☐
What does the Sámi parliament mean to you?	☐	☐	☐	☐

Do you consider pollution/interference in nature a threat to your Sámi way of life?

☐ To a large extent ☐ To some extent
☐ To a small extent ☐ Absolutely not

Do you feel that modern development displaces Sámi culture?

☐ To a large extent ☐ To some extent
☐ To a small extent ☐ Absolutely not

Thank you for your help!
Remember to post the questionnaire today!
